# Infected Anterior Mediastinal Epidermoid Cyst Mimicking Mature Cystic Teratoma and Adherent to the Pericardium: A Case Report

**DOI:** 10.1159/cro/abzag015

**Published:** 2026-08-17

**Authors:** Youssef Abbas, Mohammad Alaa Aldakak, Rahma Al-Jawalah, Yamama Abo Dakka, Raghad Mahmoud, Asmaa Dandashy, Hussain Chaban

**Affiliations:** a National University Hospital, Faculty of Medicine, Damascus University, Damascus, Syrian Arab Republic; b Faculty of Medicine, Damascus University, Damascus, Syrian Arab Republic

**Keywords:** Epidermoid cyst, Anterior mediastinum, Mediastinal cyst, Mature cystic teratoma, Video-assisted thoracoscopic surgery

## Abstract

**Introduction:**

Mediastinal epidermoid cysts are exceptionally rare benign lesions, particularly in the anterior mediastinum. Their nonspecific clinical and radiological features may resemble other anterior mediastinal masses, including thymic lesions and mature cystic teratomas, making preoperative diagnosis challenging.

**Case Presentation:**

A 35-year-old man with no relevant medical or surgical history presented with a 2-month history of retrosternal chest pain. Clinical examination and cardiac evaluation were unremarkable. Chest radiography demonstrated mediastinal widening, while contrast-enhanced computed tomography (CT) revealed a well-defined anterior mediastinal mass measuring approximately 7 × 5 × 3 cm. CT-guided biopsy and fluid aspiration initially suggested a mature cystic teratoma without immature or malignant elements. The patient underwent complete excision using a three-port video-assisted thoracoscopic approach. Intraoperatively, the cyst was densely adherent to the pericardium and contained thick purulent material. Hair-like material and firm cartilage-like areas were observed grossly but were not confirmed histologically. Culture of the cyst contents yielded *Staphylococcus aureus*. Histopathological examination demonstrated a squamous epithelial lining with chronic inflammation, granulation tissue, and fibrosis, confirming an infected mediastinal epidermoid cyst. The discrepancy between the initial cytological impression and the final histopathological diagnosis highlights the limitations of imaging and needle biopsy in evaluating rare cystic mediastinal lesions.

**Conclusion:**

Complete surgical excision provides both definitive diagnosis and effective treatment. Epidermoid cysts should be considered in the differential diagnosis of cystic anterior mediastinal masses. Video-assisted thoracoscopic excision may be feasible in carefully selected patients, even in the presence of dense pericardial adhesions.

## Introduction

Mediastinal masses are uncommon and comprise a heterogeneous group of lesions with diverse clinical, radiological, and pathological characteristics. Their diagnostic evaluation requires integration of patient age and sex, clinical presentation, imaging findings, and precise mediastinal compartmental location [1]. In the anterior mediastinum, reported distributions include thymic tumors in approximately 35% of cases, lymphomas in 25%, germ cell tumors in 20%, endocrine tumors in approximately 15%, and benign thymic lesions in 5% [1].

Mediastinal cysts are well-defined, epithelium-lined, fluid-containing lesions, and benign cystic lesions account for approximately 12–20% of all mediastinal tumors [2]. The principal cystic differential diagnoses include bronchogenic, esophageal duplication, pleural, pericardial, and thymic cysts [2]. Epidermoid cysts are exceptionally uncommon in the mediastinum, particularly in the anterior compartment, and their definitive diagnosis is frequently unexpected [3]. Histologically, they are lined by stratified squamous epithelium containing a granular layer and keratinous material. The absence of skin appendages, including hair follicles and sebaceous glands, distinguishes epidermoid cysts from dermoid cysts [3].

The clinical and radiological features of anterior mediastinal and thymic epidermoid cysts are nonspecific. Reported presentations range from incidental detection to chest pain, dyspnea, fever, or hemoptysis, while their imaging appearance may overlap with thymic hyperplasia, mature teratoma, thymic neoplasms, and lymphoma [4]. Consequently, surgical resection may be necessary to establish a definitive diagnosis [4]. A 2022 literature review identified only seven reported thymic epidermoid cysts worldwide, underscoring the rarity of this entity and the limited characterization of its imaging features [5]. In one previously reported case, an epidermoid cyst was radiologically mistaken for a left-sided subpulmonic effusion and was subsequently found to be an anterosuperior mediastinal cyst attached to the pericardium [6].

Here, we present a case of an infected anterior mediastinal epidermoid cyst that mimicked a mature cystic teratoma and was densely adherent to the pericardium in an adult man. This case report was prepared in accordance with the online supplementary CARE checklist (for all online suppl. material, see https://doi.org/10.1159/cro/abzag015) [7].

## Case Presentation

A 35-year-old man with no significant medical or surgical history and a smoking history of 15 pack-years presented with a 2-month history of retrosternal chest pain. He denied dyspnea, fever, weight loss, or other systemic symptoms. Clinical examination was unremarkable, with normal respiratory rate, pulse rate, and oxygen saturation. Initial cardiac evaluation revealed no abnormalities. Chest radiography demonstrated mediastinal widening, prompting further evaluation with contrast-enhanced computed tomography (CT), which showed a well-defined anterior mediastinal mass measuring approximately 7 × 5 × 3 cm (Fig. 1 and 2).

**Fig. 1.**
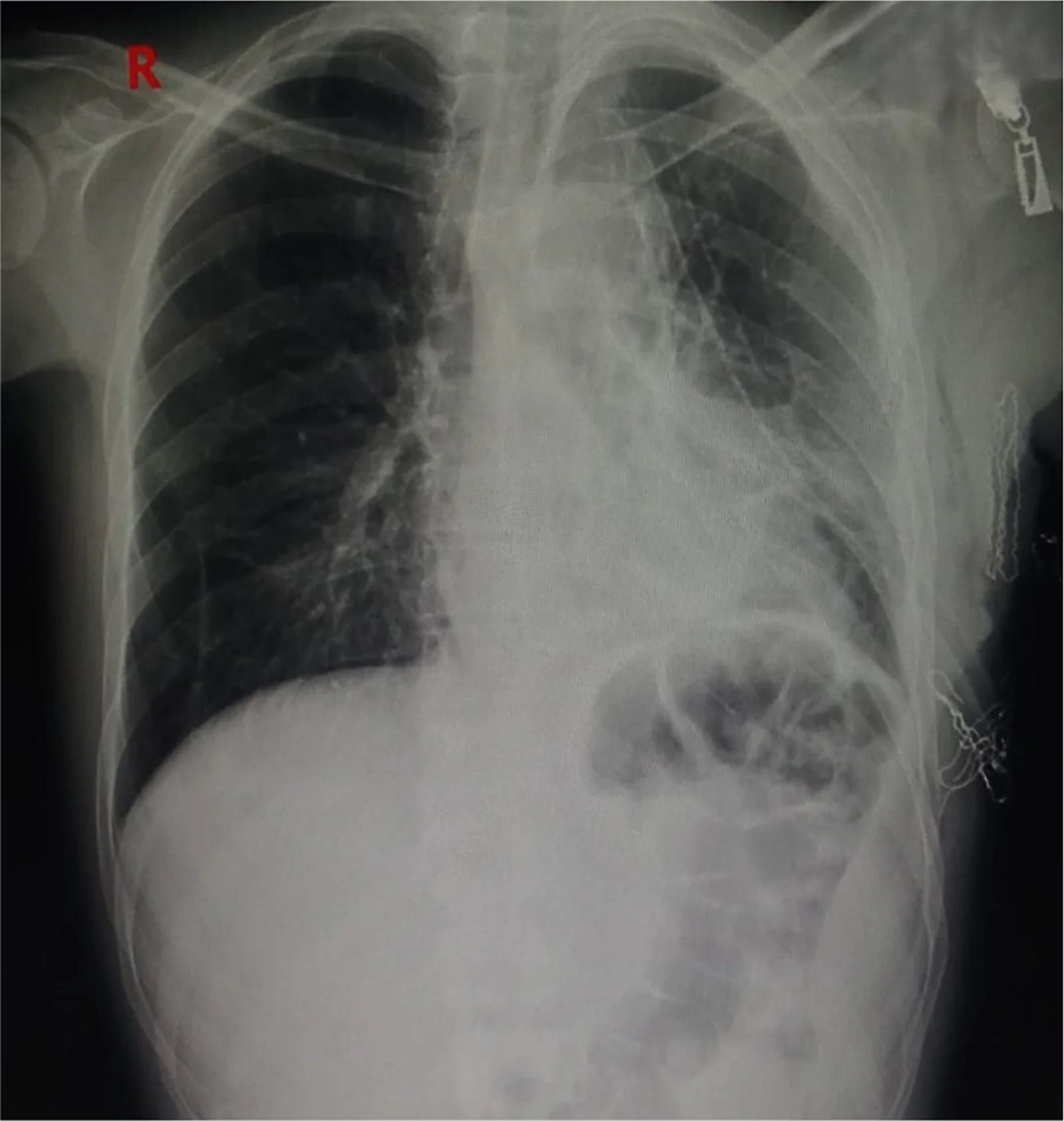
Preoperative chest radiograph. Posteroanterior chest radiograph showing mediastinal widening, prompting further evaluation with contrast-enhanced CT.

**Fig. 2.**
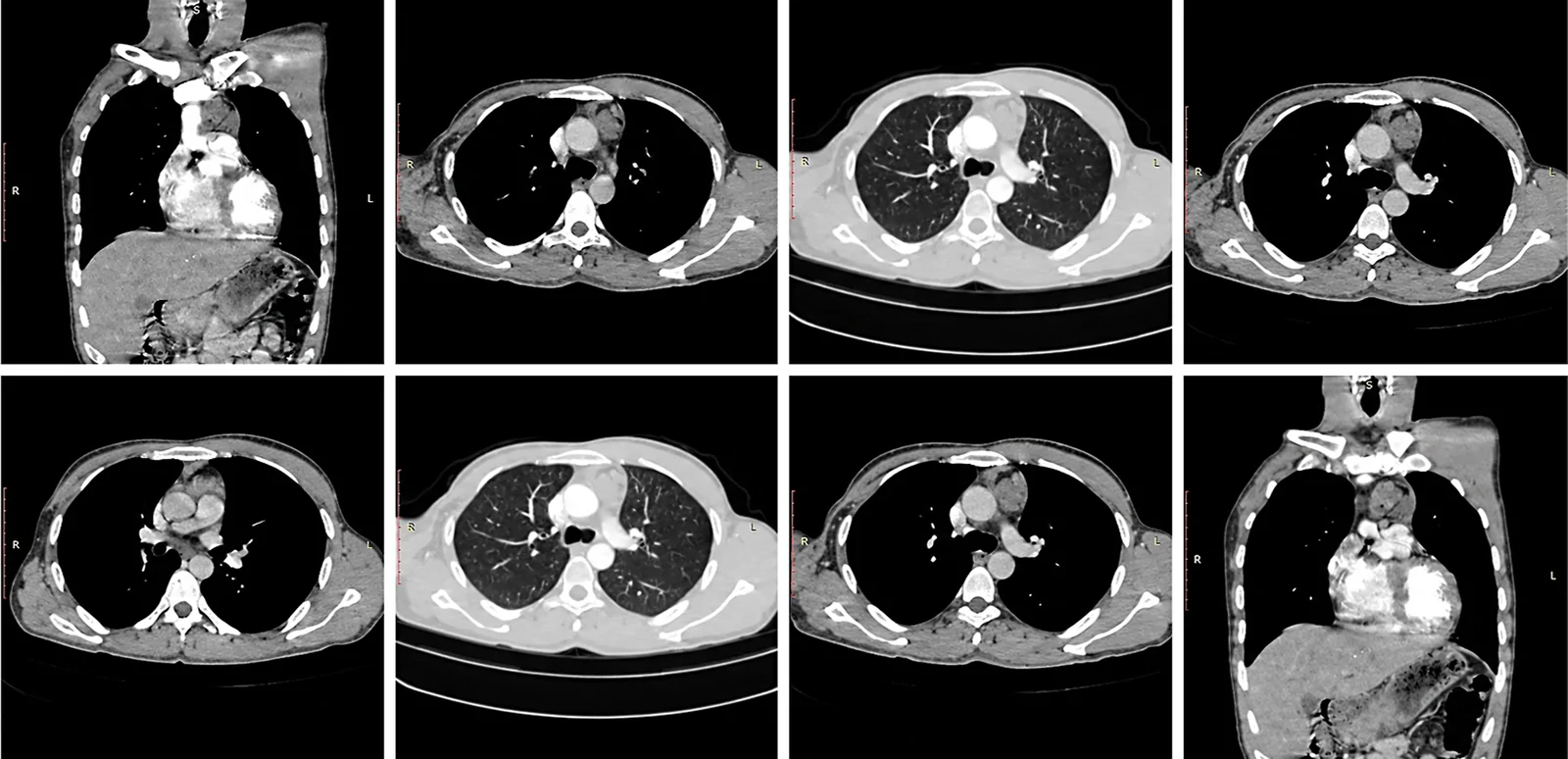
Contrast-enhanced chest CT. Axial and coronal contrast-enhanced CT images demonstrating a well-defined anterior mediastinal mass measuring approximately 7 × 5 × 3 cm, located adjacent to the great vessels and pericardium, without obvious pulmonary parenchymal invasion.

CT-guided needle biopsy and fluid aspiration were performed. Cytological examination was initially suggestive of a mature cystic teratoma, with no evidence of immature elements or malignant transformation. The submitted material consisted of soft tissue cores measuring up to 1.5 × 0.1 cm, together with 20 mL of turbid fluid and 3 mL of greasy material. Based on the clinical, radiological, and cytological findings, surgical excision was planned.

Surgery was performed 11 days after the biopsy, under general anesthesia. The patient was placed in the right lateral decubitus position with the body elevated by approximately 45°. The left pleural cavity was accessed through a three-port video-assisted thoracoscopic surgery (VATS) approach. A large cystic mass was identified in the anterior mediastinum, protruding through the left mediastinal pleura. The cyst, which was densely adherent to the pericardium, was carefully dissected thoracoscopically.

Upon controlled opening of the cyst, thick purulent material was encountered, and samples were collected for microbiological culture and antimicrobial susceptibility testing. Material grossly resembling hair and firm areas that appeared cartilage-like were also observed intraoperatively. However, these findings represented macroscopic impressions and were not corroborated by histopathological examination. The cyst contents were completely evacuated, and the entire visible cyst wall was excised (Fig. 3). Thorough irrigation and meticulous hemostasis were performed, and a 32-Fr chest tube was inserted.

**Fig. 3.**
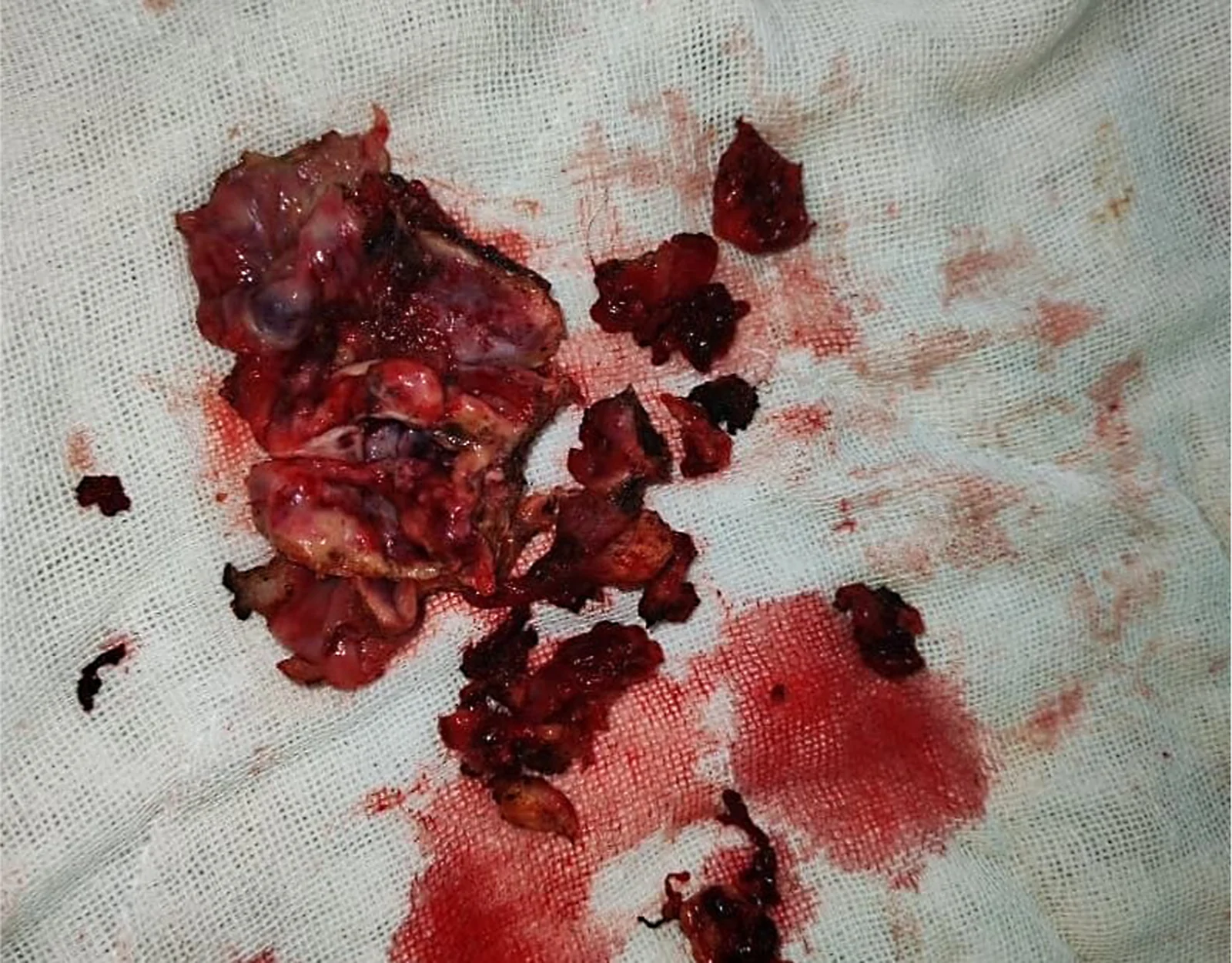
Gross surgical specimen. Gross appearance of the resected cyst wall and fragmented cystic components following complete thoracoscopic excision.

Microbiological culture of the cyst contents yielded *Staphylococcus aureus*. Antimicrobial susceptibility testing demonstrated resistance to oxacillin and cefoxitin. Postoperative antimicrobial therapy consisted of intravenous linezolid 600 mg every 12 h and intravenous meropenem 1 g every 8 h for a total of 10 days. The first 2 days of treatment were administered during hospitalization, and the remaining 8 days were completed at home.

Histopathological examination revealed multiple cyst wall fragments measuring 7.5 × 1.5 cm in aggregate, with the largest fragment measuring 6.5 × 4 × 1.5 cm. The cut surface was white-tan with focal hemorrhagic areas, and the wall thickness ranged from 0.2 to 1 cm. Microscopically, the cyst was lined by squamous epithelium and showed chronic inflammation, granulation tissue formation, and fibrosis (Fig. 4). No histological dermoid or teratomatous components were reported. These findings supported the final diagnosis of an infected mediastinal epidermoid cyst.

**Fig. 4.**
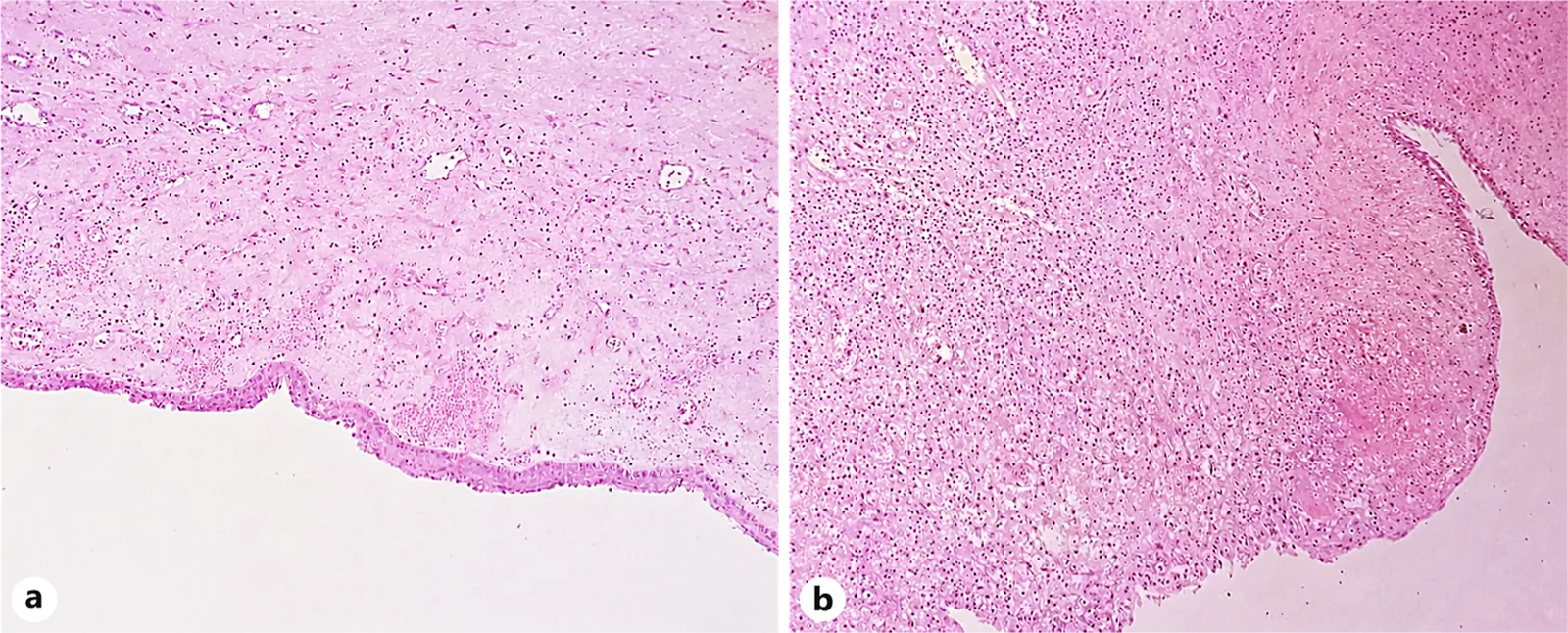
Histopathological features of the mediastinal epidermoid cyst. **a** Hematoxylin and eosin-stained section showing the cyst wall lined by squamous epithelium with underlying fibrotic tissue. **b** Hematoxylin and eosin-stained section demonstrating associated chronic inflammatory changes, granulation tissue formation, and fibrosis, consistent with an infected epidermoid cyst.

The postoperative course was uneventful. The chest tube was removed 48 h after surgery, and the patient was discharged on postoperative day 2 in good clinical condition. Postoperative chest radiography demonstrated satisfactory lung expansion without apparent pneumothorax or pleural collection (Fig. 5). At the 1-month postoperative follow-up, the patient remained clinically well and asymptomatic, with no postoperative complications or clinical evidence of recurrence. The clinical timeline is presented in Table 1.

**Fig. 5.**
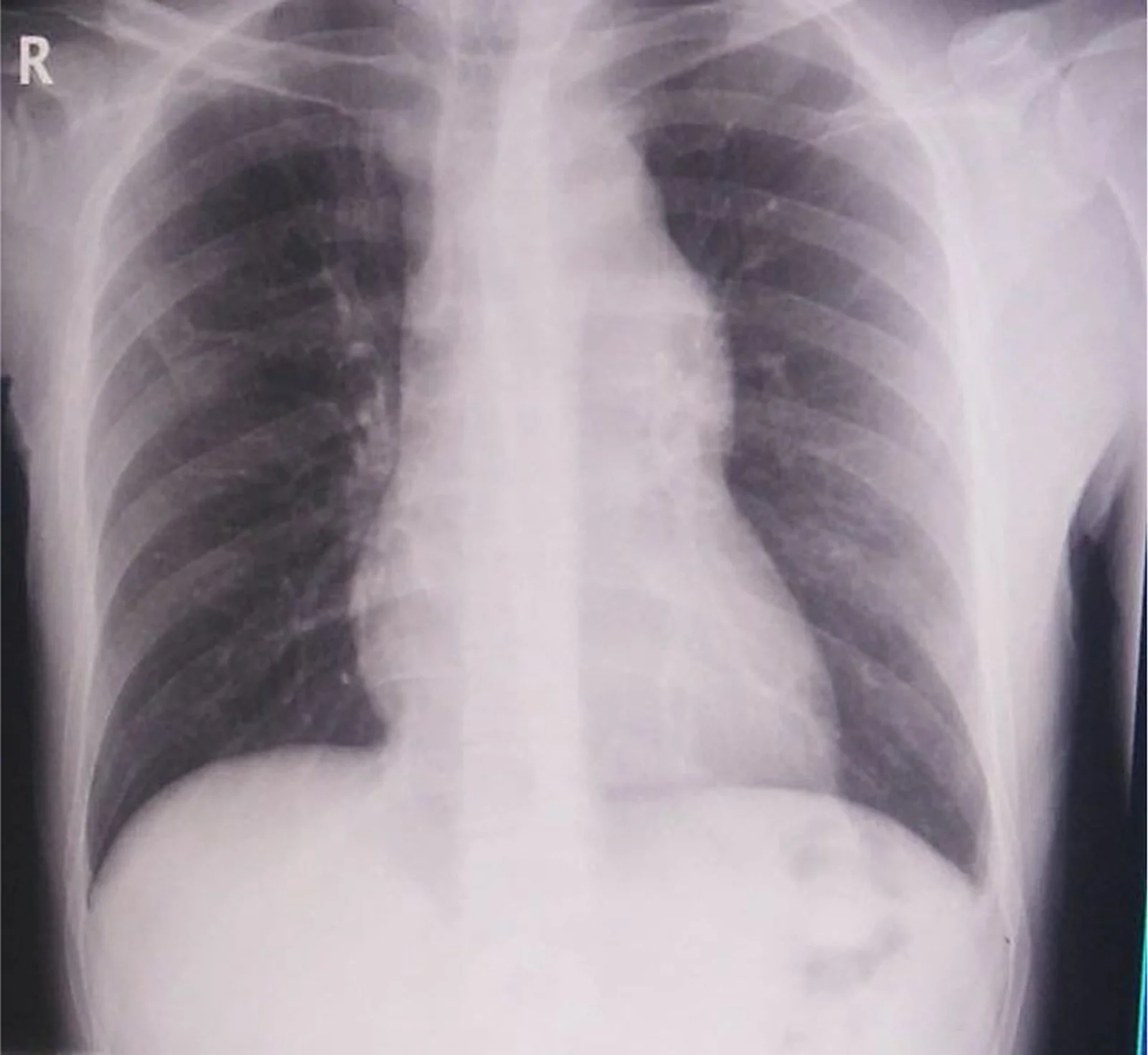
Postoperative chest radiograph. Postoperative chest radiograph obtained after video-assisted thoracoscopic excision, showing satisfactory lung expansion without apparent pneumothorax or pleural collection.

**Table 1.** Clinical timeline

Time point	Clinical event
Approximately 2 months before presentation	Onset of persistent retrosternal chest pain without dyspnea, fever, weight loss, or other systemic symptoms
Initial clinical evaluation	Physical examination and cardiac assessment were unremarkable. Chest radiography demonstrated mediastinal widening, and contrast-enhanced CT showed a well-defined anterior mediastinal mass measuring approximately 7 × 5 × 3 cm
February 5, 2026	CT-guided needle biopsy and fluid aspiration were performed. Cytological examination was initially suggestive of a mature cystic teratoma, with no immature elements or malignant transformation identified
February 16, 2026	Three-port VATS was performed. The cyst was densely adherent to the pericardium and contained thick purulent material. After controlled evacuation, the entire visible cyst wall was excised thoracoscopically
Postoperative microbiological and pathological assessment	Culture of the cyst contents grew *Staphylococcus aureus*. Histopathological examination showed squamous epithelial lining with chronic inflammation, granulation tissue, and fibrosis, supporting the diagnosis of an infected mediastinal epidermoid cyst
February 16–26, 2026	Intravenous linezolid 600 mg every 12 h and meropenem 1 g every 8 h were administered for 10 days. The first 2 days were completed in hospital and the remaining 8 days at home
Postoperative day 2 (February 18, 2026)	The chest tube was removed after 48 h. The patient was discharged in good clinical condition after postoperative chest radiography confirmed satisfactory lung expansion without apparent complications
One-month postoperative follow-up	The patient remained clinically well and asymptomatic, with no postoperative complications or clinical evidence of recurrence. No follow-up cross-sectional imaging was available

CT, computed tomography; VATS, video-assisted thoracoscopic surgery.

## Discussion

Mediastinal epidermoid cysts are exceedingly uncommon, particularly in the anterior mediastinum. Their rarity, combined with nonspecific clinical and imaging findings, makes preoperative diagnosis difficult. Accurate evaluation of an anterior mediastinal mass requires integration of the patient’s age and sex, clinical presentation, anatomical compartment, and tissue characteristics on imaging [1, 8]. This is particularly relevant in young adults because mature teratoma and other mediastinal germ cell tumors are important diagnostic considerations in this age group [1, 8, 9]. As of 2022, seven thymic epidermoid cysts had been reported worldwide [5], while a small number of additional anterior mediastinal epidermoid cysts attached to the pericardium had also been described [3, 6, 10].

The clinical presentation is variable. Some mediastinal epidermoid cysts have been detected incidentally, including the posterior mediastinal lesion reported in a child by Sloan and Lakhoo and the asymptomatic anterior mediastinal cyst reported by Nalladaru et al. [2, 3]. Other patients presented with chest pain, dyspnea, fever, or severe compressive manifestations [4–6, 10]. Sameh et al. [6] reported a large anterosuperior mediastinal epidermoid cyst presenting with chest pain and initially interpreted as a left-sided subpulmonic effusion. Sharma et al. [10] described a patient with progressive dyspnea, hypotension, superior vena cava compression, and right-sided cardiac chamber compression caused by a large cyst initially diagnosed as a loculated pericardial effusion. In comparison, our patient presented with isolated retrosternal chest pain without fever, dyspnea, weight loss, or other systemic manifestations.

Preoperative diagnosis remains challenging because the radiological characteristics of epidermoid cysts overlap with those of other cystic and solid mediastinal lesions. Previous cases have been interpreted as bronchogenic cysts, subpulmonic effusions, loculated pericardial collections, complicated cystic lesions, or thymic neoplasms [2, 4–6, 10]. In the present case, CT-guided biopsy and aspiration initially suggested a mature cystic teratoma. This interpretation was understandable because mature mediastinal teratomas commonly occur in young adults and may contain cystic components, fat, calcification, hair, cartilage, or sebaceous material [1, 8, 9]. Moreover, the aspirated greasy material and the intraoperative appearance of hair-like and firm cartilage-like structures reinforced the initial impression of a teratomatous lesion.

Nevertheless, the intraoperative findings represented gross macroscopic impressions and were not corroborated by histopathological examination. The resected cyst wall demonstrated squamous epithelial lining with chronic inflammation, granulation tissue formation, and fibrosis. No histological dermoid or teratomatous components were reported. A typical epidermoid cyst is lined by stratified squamous epithelium with a granular layer and contains keratinous material, whereas dermoid cysts contain skin appendages such as hair follicles and sebaceous glands [2, 3, 5, 10]. Mature teratomas, in contrast, contain tissues derived from more than one embryonic germ layer [1, 8]. This discrepancy between the gross appearance and definitive microscopy emphasizes that limited cytological or aspirated material may be misleading and that examination of the resected cyst wall remains essential for diagnosis.

A distinctive feature of the present case was superimposed infection. Thick purulent material was encountered intraoperatively, tissue culture yielded *S. aureus*, and histopathological examination demonstrated prominent inflammatory, granulation, and fibrotic changes. The precise mechanism of infection could not be established. CT-guided biopsy and aspiration had been performed 11 days before surgical excision. Although infection may have been present before the procedure, secondary bacterial inoculation following percutaneous biopsy and aspiration cannot be excluded. The temporal association and isolation of *S. aureus* make this mechanism plausible, but they do not establish a definitive causal relationship.

The close relationship with the pericardium is consistent with several previous reports. In the case reported by Nalladaru et al. [3], the anterior mediastinal cyst was attached to the right side of the pericardium and was excised through an anterior thoracotomy. Sameh et al. [6] identified a large anterosuperior mediastinal cyst attached to the pericardium during thoracotomy. Sharma et al. [10] similarly reported a large pericardium-attached cyst causing marked compression of the right atrium, right ventricle, and superior vena cava. Our case shared the anterior location and pericardial relationship of these reports but was characterized by dense inflammatory adhesion rather than compression alone.

Surgical resection has both diagnostic and therapeutic roles, particularly when malignancy or a germ cell tumor cannot be confidently excluded [3–5, 11]. Open thoracotomy or median sternotomy was used in several previously reported anterior mediastinal cases [3, 6, 11]. In the pediatric posterior mediastinal case, an initially thoracoscopic procedure required conversion to thoracotomy because of adherence to the inferior vena cava and diaphragm [2]. Conversely, minimally invasive thoracoscopic or robotic resection has been successfully performed in selected thymic epidermoid cysts without extensive involvement of adjacent structures [4, 5]. In our patient, the cyst was completely dissected thoracoscopically despite dense pericardial adhesion, without conversion to thoracotomy or apparent perioperative complications.

This case therefore suggests that VATS may remain feasible in carefully selected patients with pericardial adhesions, provided that meticulous dissection can be performed safely and that conversion to an open approach remains available if a safe plane cannot be maintained. However, the findings from a single case should not be generalized to all infected or adherent anterior mediastinal epidermoid cysts. Additional reports and larger case series are needed to better define appropriate patient selection, operative risks, and the comparative role of VATS and open surgery in lesions complicated by infection or dense adherence to adjacent mediastinal structures.

This report has several limitations. First, it describes a single patient, limiting the generalizability of conclusions regarding thoracoscopic management. Second, the precise mechanism and timing of infection could not be definitively established, and secondary inoculation following CT-guided biopsy cannot be excluded. Third, the intraoperative hair-like and cartilage-like appearances were not confirmed histologically. Finally, postoperative follow-up was limited to 1 month and did not include cross-sectional imaging; therefore, longer clinical and radiological follow-up would be required to more confidently exclude residual disease or recurrence.

## Conclusion

Mediastinal epidermoid cysts are extremely rare, particularly in the anterior mediastinum, and their preoperative diagnosis is challenging because they may mimic other cystic lesions, including mature cystic teratoma. In the present case, the cyst was infected, densely adherent to the pericardium, and initially interpreted as a mature cystic teratoma, whereas definitive histopathological examination established the diagnosis of an epidermoid cyst. Complete surgical excision is generally recommended for both diagnostic and therapeutic purposes. This case emphasizes the importance of considering an epidermoid cyst in the differential diagnosis of anterior mediastinal cystic masses and suggests that complete thoracoscopic excision may be feasible in carefully selected patients, even in the presence of dense pericardial adhesions.

## Statement of Ethics

The Ethics Committee at Damascus University does not require ethical approval for reporting individual cases or case series. Written informed consent was obtained from the patient for publication of this case report and any accompanying images. The CARE Checklist for this case report is available as online supplementary material

## Conflict of Interest Statement

The authors have no conflicts of interest to declare.

## Funding Sources

This study was not supported by any sponsor or funder.

## Author Contributions

Youssef Abbas contributed to the clinical management of the patient, interpretation of surgical findings, and critical revision of the manuscript for important clinical content. Mohammad Alaa Aldakak collected the patient’s clinical data, performed the literature review, drafted the initial manuscript, and contributed to manuscript revision and final editing. Rahma Al-Jawalah contributed to patient data collection, literature review, manuscript preparation, and revision of the case presentation. Yamama Abo Dakka assisted in patient monitoring, coordinated the diagnostic workup, provided clinical information, and reviewed the manuscript. Raghad Mahmoud contributed to literature review, manuscript editing, and formatting of the final draft. Asmaa Dandashy performed the histopathological evaluation of the excised specimen, contributed to the pathological diagnosis, and critically reviewed the manuscript for pathology-related content. Hussain Chaban supervised the overall clinical management of the case and manuscript development, contributed to the interpretation of clinical and surgical findings, ensured scientific accuracy, and critically reviewed the final version of the manuscript. All authors have read and approved the final version of the manuscript.

## Data Availability

All data generated or analyzed during this study are included in this article and its online supplementary material. Further inquiries can be directed to the corresponding author.
